# Construction of Isotropy-Enhanced Honeycomb and Its Deformation Behaviors in Multi-Directions

**DOI:** 10.3390/polym17121717

**Published:** 2025-06-19

**Authors:** Junyuan Zheng, Guangdong Tian

**Affiliations:** School of Mechanical-Electronic and Vehicle Engineering, Beijing University of Civil Engineering and Architecture, Beijing 100044, China; zhengjunyuan@bucea.edu.cn

**Keywords:** hexagonal honeycomb, out-of-plane, in-plane, energy absorption, isotropic mechanical properties

## Abstract

Honeycomb structures are widely constructed as cores in sandwich panels with lightweight characteristics and excellent out-of-plane properties. However, their in-plane performances are significantly inferior. This research proposed a novel isotropy-enhanced honeycomb (IEH) with interleaved layers, which is constructed by offsetting the initial seed distributions across layers and then generating hexagonal cells via Voronoi tessellation. Numerical models with three layer-to-layer interval gradients were developed for simulations, and corresponding samples were additively manufactured for experimental validations. The in-plane and out-of-plane performances of IEH and the regular hexagonal honeycombs (RHHs) were comprehensively compared and investigated from quasi-static compression, energy absorption, mechanical properties, and dynamic loading. The results demonstrated that the IEH extremely enhances the in-plane properties by around 500% compared to the RHH, including stiffness, strength, plateau stress, and specific energy absorption (SEA). Although the improvements come at the expense of a partial reduction in out-of-plane stiffness, strength, and SEA, the in-plane performances of IEH reach approximately 70% of their out-of-plane performances, greatly improving the structural isotropy. Introducing layer-to-layer interval gradient leads to a slight reduction in out-of-plane mechanical properties while improving the early-stage deceleration under impact. These findings promote the considerable potential of sandwich panels utilizing IEH cores for applications requiring enhanced resistance to multi-directional impacts.

## 1. Introduction

Cellular structures have garnered significant attention due to their lightweight and multifunctional properties for their potential and promising applications in fields such as energy absorption [1], heat dissipation [2], and noise and vibration control [3], etc. In recent years, a variety of bio-inspired cellular structures have been developed and thoroughly investigated from perspectives including strength, energy absorption capability, and distinctive characteristics, such as triply periodic minimal surface (TPMS) [4,5], origami [6], lattice [7,8], and honeycomb [9,10]. The mechanical properties of these cellular structures, such as stiffness, initial crushing stress, subsequent plateau stress, and densification strain, are critically influenced by their underlying architecture and relative density [11]. In particular, the honeycomb could exhibit special properties like auxetic [12,13], chiral and anti-chiral [14], hierarchical [15], or high stiffness [16] owing to its distinct features. By introducing a density gradient, the honeycomb can also obtain a progressively crushing deformation mechanism [17]. Meanwhile, growing interests have been driven by the development of additive manufacturing (AM) processes, also known as 3D printing, including fused deposition modeling (FDM), selective laser melting (SLM), stereo-lithography apparatus (SLA), and so on [18].

Honeycomb structures can achieve tunable stiffness and Poisson’s ratio by adjusting the design configurations and the shape of the cross-section. Researchers have conducted extensive studies on their unique geometrical characteristics and mechanical performances. Murray et al. [19] found that the buckling imitators located on honeycomb walls can increase energy absorption efficiency regardless of size. Zhang et al. [20] designed an origami-inspired honeycomb with a negative Poisson ratio, which enhanced the strength and energy absorption capacity for optimized cushioning. Yu et al. [21] developed a 3D sinusoid–parallel hybrid honeycomb, underpinned by the elastic jumping negative Poisson ratio, showing remarkable energy absorption and impact resistance. Hou et al. [22] developed a flexible electrode with negative-Poisson-ratio honeycombs, improving the structural fatigue performance by 18 times. Chibar et al. [23] used adjustable-stiffness honeycomb metamaterials for tactile sensors, demonstrating that altering the honeycomb in a dynamic manner can effectively reduce the maximum impact force. Xiao et al. [24] constructed a butterfly-inspired honeycomb with a Poisson ratio of zero, achieving low tangential equivalent stiffness to avoid out-of-plane warping. Pei et al. [25] designed an underwater re-entrant multiphase honeycomb with excellent broadband water-like acoustic properties and impedance matching, reducing the sound speeds by half.

Honeycomb structures commonly serve as core materials in sandwich panels, primarily to withstand out-of-plane loads. Prior research made efforts to develop innovative design configurations to optimize the out-of-plane performances and to investigate the differences between the in-plane and out-of-plane mechanical properties of honeycombs. Tao et al. [26] developed a circular-vertex-based hierarchical hexagonal honeycomb, showing enhanced damage resistance and strength due to their geometrical features. Cecchini et al. [27] optimized the performances of honeycomb sandwich structures under quasi-static three-point bending, revealing that cell wall angle and skin thickness promote flexural stiffness. Wei et al. [28] developed a star-triangular honeycomb for in-plane energy absorption. It was found that wall thickness and inclination significantly impact the dynamic response and energy absorption at medium- and low-speed crushing. Wang et al. [29] developed a re-entrant star-shaped honeycomb for in-plane impact resistance, noting its transverse contraction within the first plateau region for better mechanical response. Alomarah et al. [30] designed a re-entrant chiral auxetic honeycomb, exhibiting superior out-of-plane energy absorption under quasi-static uniaxial compression. Dong et al. [31] developed a biomimetic modular honeycomb, indicating that adjusting the wall thickness can improve the resistance to low-velocity compressive impacts. Han et al. [32] determined the elastic modulus of nanoporous hierarchical honeycombs via nanoindentation and uniaxial compression simulations and developed the macroscopic stress–strain relationship. Wang et al. [33] established a continuous solid equivalent honeycomb model, revealing that compressive strength is decreased with increasing off-axis angles due to the anisotropic behavior, strain rate, and tearing effect.

The aforementioned research demonstrated the diversity of characteristics of honeycomb structures and their robust out-of-plane mechanical performances, making them highly promising for the requirements of a high strength-to-weight ratio. However, the significant disparity in load-bearing strength between the in-plane and out-of-plane directions induces their limitations in cushioning protection. Therefore, a novel honeycomb with interleaved layers, integrated with multi-level hexagonal honeycombs, was proposed, which has an enhanced isotropy compared to the regular hexagonal honeycomb (RHH); thus, it is named isotropy-enhanced honeycomb (IEH). The IEH fundamentally alters the cellular shape compared to the RHH, introducing transverse bearing walls between the neighboring layers, which is similar to origami structures. These walls are nearly aligned with the in-plane direction, thus enhancing the in-plane strength of structures. However, the out-of-plane bearing walls in IEH are misaligned compared to RHH due to the multi-level design, decreasing the out-of-plane strength to some extent. Furthermore, the IEH structures with three layer-to-layer interval gradients are additively manufactured for experiments. Quasi-static compression tests along the building direction, lateral direction, and transverse direction corresponding to finite element (FE) simulations were conducted with in situ photography. The in-plane and out-of-plane compressive responses of IEH were analyzed and compared with RHH, and their specific energy absorption and energy absorbing efficiency were evaluated. Meanwhile, the mechanical properties including elastic modulus, yield stress, and average plateau stress of IEH and RHH were determined, and their differences in two typical directions were discussed. Moreover, the deformation mechanism and crushing behavior were analyzed according to the stress distribution in simulations, validated by in situ photos during compression tests. In addition, the response of IEH under instantaneous loading was detected by FE simulations to investigate the effect of layer-to-layer interval. This work provides new insights into the design of honeycomb structures and promotes isotropic performances in multi-directional shocks, broadening the cushioning and energy absorption applications in complex loading conditions.

## 2. Materials and Methods

### 2.1. Development and Fabrication of IEH Structures

**Figure 1:**
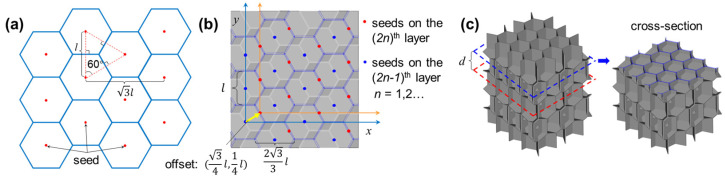
The design and development of IEH model: (**a**) the two-dimensional hexagonal patterns, where the red points are initial seeds and blue lines are Voronoi borders; (**b**) the distribution of initial seeds at odd and even layers with an offset and the critical parameters of seed distributions; (**c**) the geometries of IEH generated by Voronoi method from initial seeds with a specific distance between the neighboring layers.

The IEH structures were inspired by RHH structures. The two-dimensional RHH patterns can be designed by integrating the Voronoi tessellation [34], as shown in Figure 1a. The Voronoi region *V* can be defined as follows:

(1) $$V\left(p_{i}\right)=\{x\in R^{2}|\left\|x-p_{i}\right\|\leq \left\|x-p_{j}\right\|,\;\forall j\neq i,\;1\leq j\leq k\},$$

where $p_{i}$ is a seed belonging to a set of seeds $P=\{p_{1},p_{2},\ldots ,p_{k}\}$, and *x* is any point in the two-dimensional real number space $R^{2}$. This inequality states that all points *x* within $V\left(p_{i}\right)$ are closer to $p_{i}$ than to any other site $p_{j}$, with j≠i. Moreover, the coordinates of seed $p_{mn}$ belonging to *P* can be expressed as:

(2) $$p_{mn}=\left[m\cdot \frac{\sqrt{3}}{2}l,\left(nl+m\%2\cdot \frac{l}{2}\right)\right],\;m,n\in Z,$$

where *m* and *n* represent the sequence of seed $p_{mn}$ on the *x*- and *y*-axis, % indicates the remainder, and *l* is the distance between the neighboring seeds, respectively.

The planar hexagonal pattern can be developed through the Voronoi method by distributing seeds at specific positions, where each seed has six closest neighboring seeds. RHH structures are the extrusion of the planar hexagonal pattern along the third dimension, while IEH structures are established through periodically arranging two misaligned RHH structures. As displayed in Figure 1b,c, the interval of neighboring layers is *d*, and the distributions of seeds at the odd layers and the even layers are in the same pattern following Equation (2), but there is a deviation vector $\left(\sqrt{3}/4l,1/4l\right)$. After distributing seeds according to the specific rule, the three-dimensional Voronoi geometries were generated by using SciPy v1.15.2 in Python 3.13 library.

Furthermore, the distance between neighboring layers *d* varies by introducing a parameter *λ*, which is expressed in the following relationship:

(3) $$d=d_{0}\lambda^{k},$$

where *d* is the distance between the (*k* − 1)-th and *k*-th layers, and $d_{0}$ is the initial distance between the 1st and 2nd layers. By adjusting *λ* > 1, a gradient IEH structure can be obtained. *λ* is a gradient parameter to tailor the geometry of cells, which provides an extra design configuration for non-uniform design.

**Figure 2:**
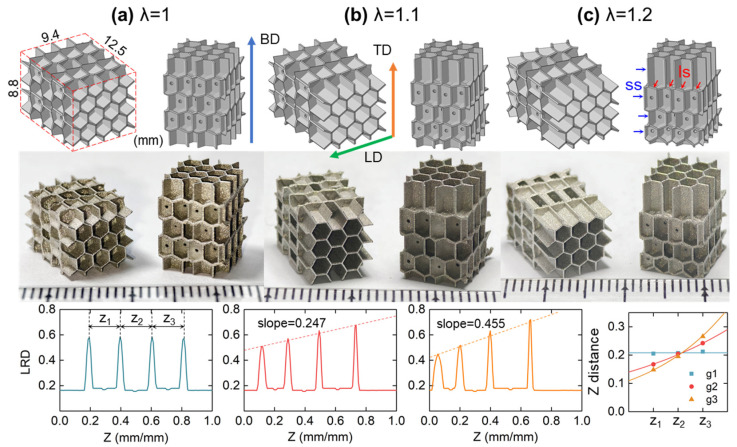
The solid model, specimen, and LRD of IEH structures with variable gradient: (**a**) *λ* = 1; (**b**) *λ* = 1.1; (**c**) *λ* = 1.2. The abbreviation “ss” refers to the supported surface, represented as blue arrows, and “ls” refers to the layered surface, represented as red arrows, respectively. The image in the bottom-right corner plots the distance between LRD peaks.

As shown in Figure 2, three types of IEH structures in the size of 8.8 × 9.4 × 12.5 mm^3^ with five interleaved layers of hexagonal features were designed and fabricated. Each specimen has the same two types of layers, and the gradient follows building direction (BD). In these IEH structures, a supported surface (ss) exists in the RHH structures, which is the main load-bearing surface under out-of-plane loading. A layered surface (ls) is newly generated by IEH development, located between the neighboring layers, which are the strengthening mechanisms under in-plane loading. To directly reflect the variation in density along the gradient direction, local relative density (LRD) is introduced to reflect the localized material density compared to the base material, which is expressed as:

(4) $$\rho_{LRD}(Z)=A_{eff}/A_{total},\;Z\in [0,1]$$

where *Z* is the relative position at the *z*-axis (BD), ranging from 0 to 1, $A_{eff}$ is the effective area at a given *Z*, and $A_{total}$ is the area occupied by geometry at a given *Z*. The relationship of LRD and *Z* of three types of IEHs is shown in Figure 2. It is revealed that the peak value appears at the transition between two types of hexagonal geometries and increases with *Z* for a higher *λ*, which is related to the cellular shape and the angle of the interface. The above parameters in various design configurations are summarized in Table 1, and the relative density *ρ_s_* is the average value of LRD, which represents the porosity of structures.

In this research, the IEH structures were additively manufactured by laser powder bed fusion (LPBF), also known as SLM. A customized micro-LPBF system was established and equipped with a continuous 50 W IPG fiber laser, whose wavelength was 1.07 µm and beam spot size was 25 µm, respectively. In the micro-LPBF process, austenitic stainless steel (SS316L) powder was used as base material, whose particle size was in the range of 5~25 µm with a $D_{50}$ value of 16.27 µm. The optimized processing parameters are summarized in Table 2, and the photos of AMed specimens are shown in Figure 2.

### 2.2. Mechanical Properties of Base Material

**Figure 3:**
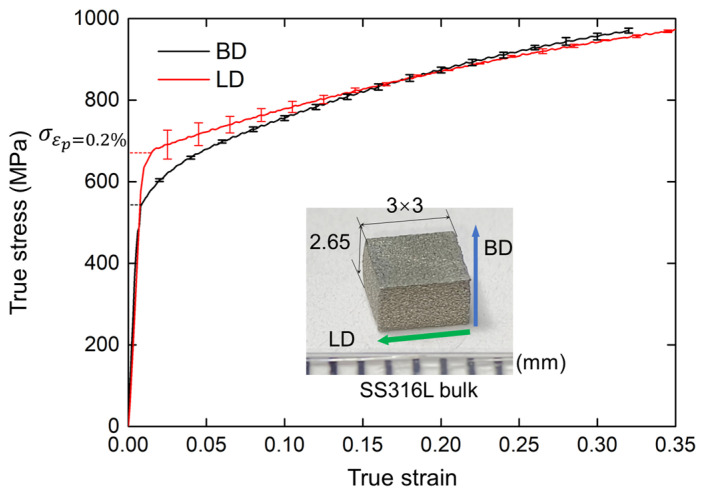
The flow stress of the LPBF-fabricated SS316L bulks along the building direction (BD, black curve) and lateral direction (LD, red curve). The dashed lines indicate the yield stress when the plastic strain reaches 0.2%.

To precisely determine the material properties of the base material, SS316L bulks in size of 3 × 3 × 2.65 mm^3^ were fabricated by the micro-LPBF system. Uniaxial compressive tests were conducted using a universal testing machine with a 50 kN load cell. It is a quasi-static process with a low stain rate of 0.001 s^−1^ at room temperature. Due to the orthotropy of SLM-fabricated components, two groups of uniaxial compressive tests along BD and lateral direction (LD) were conducted with three repeated tests, and their results are illustrated in Figure 3. It is revealed that there is a deviation between yield stress between BD and LD, where LD shows a higher stiffness; since the cylindrical grains preferred to grow along the building direction, inducing the grain size in LD is lower than in BD, and thermal residual stress remains, further resulting in the enhancement in LD of base material. It is also observed that the fracture strain (*ε_p_*) on LD is slightly larger than on BD, suggesting better plasticity. Meanwhile, the flow stress in LD and BD becomes similar when the true strain reaches around 0.15, indicating the difference is eliminated at relatively large deformation.

The mechanical properties of the SS316L bulks can be fitted into a power-law constitutive model, which is expressed as:

(5) $$\bar{\sigma }(\bar{\varepsilon })=\sigma_{y}+K{\bar{\varepsilon }}^{c},$$

where $\bar{\sigma }$ is the flow stress, $\bar{\varepsilon }$ is the plastic strain, $\sigma_{y}$ is the yield stress, determined by the point when the plastic strain reaches 0.2%, *K* is the strength coefficient, and *c* is the hardening rate, respectively. The material parameters are summarized in Table 3.

### 2.3. Experimental and Numerical Methods

**Figure 4:**
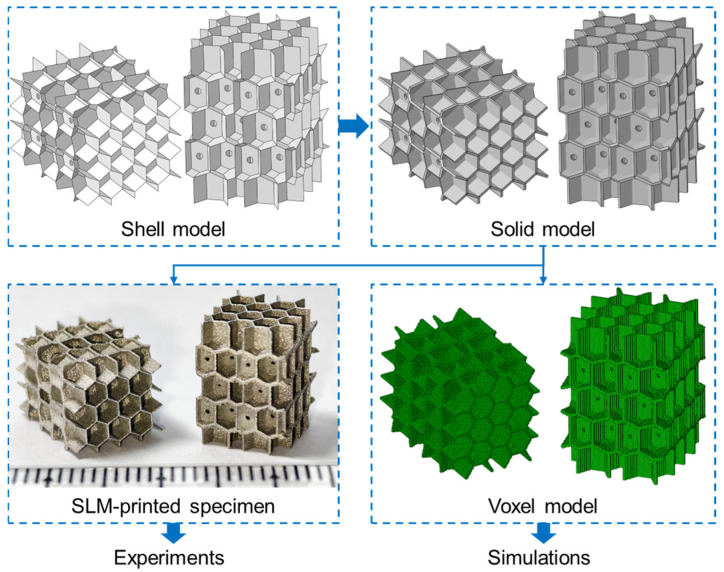
The establishment of IEH structures from shell model to solid model, then finally specimen and voxel model for experiments and simulations.

As shown in Figure 4, the shell model was first established from the Voronoi geometries, where small penetrating holes were designed for powder cleaning of fabricated parts. Next, the solid model was formed by offsetting the shell model to the normal on both sides. The shell thickness of the solid model is 0.2 mm. Then, the 3D stereo-lithography (STL) model was generated from the solid model, and specimens were fabricated by micro-LPBF based on the STL model for experimental investigations. The fabricated parts were first cleaned with an ultra-sonic cleaner to discharge the remained powder. The experimental compression tests were conducted via the universal testing machine under quasi-static conditions, and the loads and crosshead of the punch were extracted for calculation. The compression tests were conducted two times for each specimen to verify the repeatability. Meanwhile, the voxel model was generated from the STL model by using the VoxelGrid tool in the trimesh v4.6.12 in Python 3.13 library with a voxel size of 0.1 mm, which is used for simulations.

**Figure 5:**
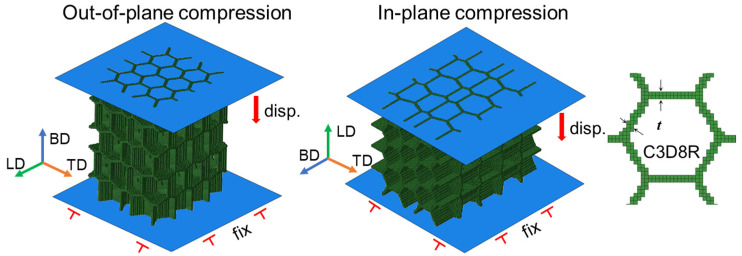
The boundary conditions and mesh type in FE simulations.

The FE simulations were conducted on the *Abaqus/Explicit 2016* platform using the voxel model generated by Python script. As shown in Figure 5, two rigid shell plates were employed on the two ends of structures, perpendicular to the movement direction, whose interfaces were generally in hard contact with a friction coefficient of 0.2. A displacement boundary condition was applied on the top movable plate to move down with a strain rate of 1 s^−1^, while the opposite one was fixed in all freedoms during the compression. Semi-automatic mass scaling was applied with a minimum target time increment of 10^−5^ s, ensuring the ratio of kinetic energy over internal energy was lower than 10% for a quasi-static deformation. The structures were meshed in 3D 8-node reduced elements (C3D8R) in size of 0.1 mm, where the number of elements in each model was around 230,000. The constitutive model and material parameters developed in Equation (3) and Table 3 were utilized in material properties.

## 3. Results

### 3.1. Compressive Response and Energy Absorption

**Figure 6:**
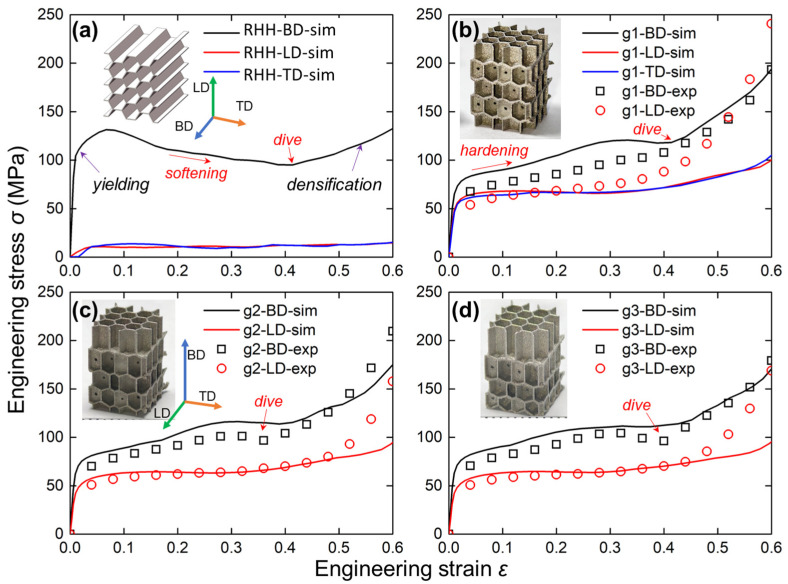
The engineering stress and strain relationships from experimental and simulation results for different structures: (**a**) The RHH; (**b**) The IEH with *λ* = 1; (**c**) The IEH with *λ* = 1.1; (**d**) The IEH with *λ* = 1.2. The curve represents the simulation results, and the scatter represents the experimental results.

**Figure 7:**
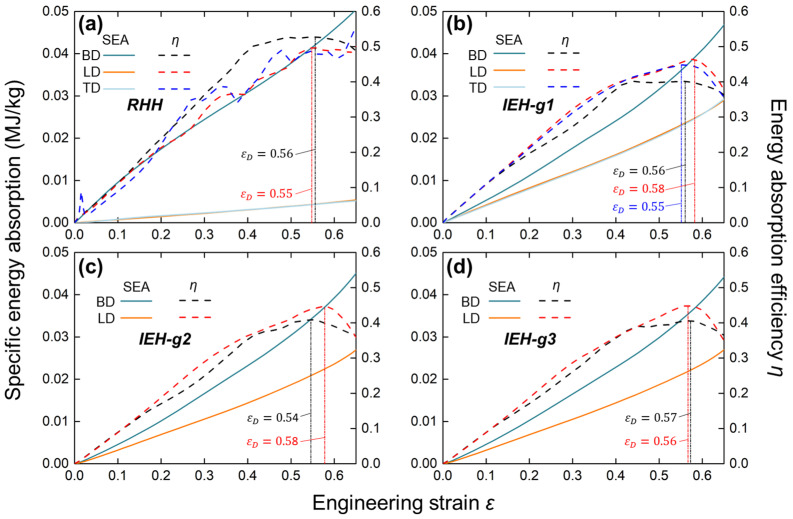
The specific energy absorption (SEA) and the energy absorption efficiency *η* with engineering strain of RHH and IEH structures obtained from simulations: (**a**) the RHH; (**b**) the IEH with *λ* = 1; (**c**) the IEH with *λ* = 1.1; (**d**) the IEH with *λ* = 1.2.

To comprehensively compare the mechanical properties of IEH and RHH structures, the engineering stress versus engineering strain curves in different structures and loading directions are shown in Figure 6. The compressive responses along three typical directions are determined, which are building direction (BD), lateral direction (LD), and transverse direction (TD). As shown in Figure 6a, the engineering stress along LD and TD is closed, and along BD it has a huge offset from the other two, indicating strong orthotropy, and a large difference between the in-plane and out-of-plane mechanical performance of RHH structures. Meanwhile, there are two critical points on the curves that need attention, which are yielding and densification. The yielding point is distinguished where the stress is increased nonlinearly, indicating the structures start to deform plastically. The stress curve shows a decreasing trend and rebounds at the later stage. However, the densification point cannot be recognized directly from curves, which are determined by energy absorption efficiency in Figure 7. Subsequently, as shown in Figure 6b, the IEH structures significantly increase the mechanical strength in both LD and TD, which means the orthotropic difference has been greatly reduced. In addition, the experimental results have good agreements with numerical results, but hardening appears earlier in experiments, suggesting the existence of undesirable geometries inside the AMed parts, which hinders the deformation. Furthermore, similar phenomena are observed in Figure 6c,d. It can be found that λ has negligible influence on the stress–strain curves since the load-bearing capacity mostly depends on the cellular shape and the surface along the loading direction. The other surfaces play a secondary role in the latter deformation stage, and their angles to the loading direction also matter. Introducing λ only changes the positions of layered surfaces and has little effect on the surface angle; thus, λ has negligible influence on the load-bearing capacity.

The specific energy absorption (*SEA*) is described by the energy absorption capability per unit mass under the uniaxial compression process. In this work, the *SEA* is expressed as follows:

(6) $$SEA=\frac{\int_{0}^{\varepsilon }\sigma \left(\varepsilon \right)d\varepsilon }{{\rho \cdot \rho }_{s}}\;,$$

where ε is the engineering strain, σ is the engineering stress, $\rho_{s}$ is the relative density of structures, and ρ is the density of SS316L. Furthermore, to better determine the optimal strain range for energy absorption of honeycomb structures, the energy absorption efficiency *η* is introduced, which is determined as:

(7) $$\eta =\frac{1}{\sigma (\varepsilon )}\int_{0}^{\varepsilon }\sigma \left(\varepsilon \right)d\varepsilon \;,$$

The comparison of *SEA* and *η* of various design configurations along multi-direction from simulation results is illustrated in Figure 7. As shown in Figure 7a, the SEA curves of the RHH structure show a huge difference between BD and the other two directions. In contrast, the SEA curves of IEH structure greatly decrease this difference, as illustrated in Figure 7b. The energy absorption capability along BD is slightly reduced, but along both LD and TD, it is significantly increased. By comparing Figure 7b–d, the gradient factor *λ* has no obvious influence on SEA. On the other hand, energy absorption efficiency *η* reflects the rate of energy absorption of structures under compression, where the maximum values of the curves indicate the onset of densification. It is revealed that densification occurs at the strain of 0.55 to 0.58, and the structures and gradients have no regular effect on the onset of densification.

### 3.2. Mechanical Properties of Structures

**Figure 8:**
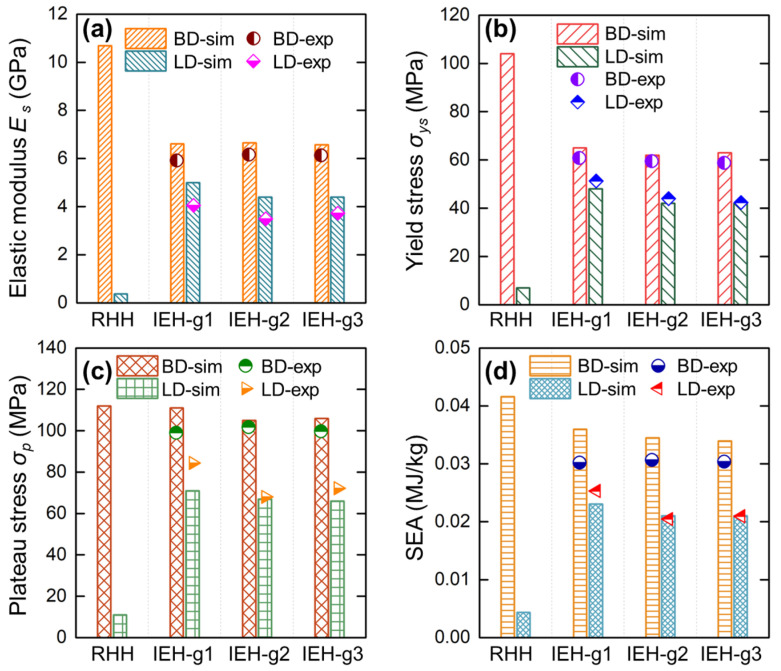
The mechanical properties of RHH and IEH with different gradient factors along BD and LD from experiments and simulations: (**a**) the elastic modulus *E_s_*; (**b**) the yield stress *σ_ys_*; (**c**) the plateau stress *σ_p_*, which is the mean engineering stress from yielding to densification; (**d**) SEA at the strain of 0.55.

To comprehensively investigate the enhanced performances of IEH compared with RHH, the mechanical properties along both BD and LD measured from experiments and simulations are summarized in Figure 8. It is shown that the experimental results are fairly consistent with the simulation results. To focus on the impact of design while minimizing the influence of experimental variability, the subsequent analysis and discussion are based on the simulation results. As shown in Figure 8a, the elastic modulus along BD is reduced by 38% from RHH to IEH, but along LD, it is greatly increased by over 1200%, indicating a significant enhancement in structurally isotropic stiffness. Meanwhile, by introducing gradient (IEH g2 and g3), the elastic modulus along LD is slightly diminished. The yield stress with different design configurations along two directions is plotted in Figure 8b. In contrast with RHH, the yield stress of IEH is reduced by 37% along BD and increased by 585% along LD, similar to the variation in elastic modulus. These results indicate that the IEH structures have achieved isotropic resistance of elastic deformation in multiple loading directions. Moreover, the plateau stress, which is the average value of stress during the stable deformation stage of structures (from yielding to densification), reflects the structural performance during the main energy absorption process. The plateau stress of RHH and IEH structures along two directions is displayed in Figure 8c. It is revealed that the plateau stress of RHH and IEH structures in BD is similar, indicating the IEH structure loses little performance along BD during the major energy absorption process. However, the plateau stress of IEH along LD is obviously enhanced by 536% compared to RHH, indicating that IEH structures are capable of effectively absorbing energy in all directions. Finally, to quantitively compare the energy absorption capability of structures, the SEA at a strain of 0.55 for various design configurations along BD and LD is shown in Figure 8d. The results show that the SEA of IEH structures along BD decreased by around 13%, but this value along LD greatly increased by 430%. Moreover, the mechanical properties and energy absorption of IEH structures with gradient design are slightly diminished.

Overall, the IEH structures are partially deteriorated in the perspective of stiffness and strength along BD, but these properties are excessively increased along LD. During the primary deformation process, the plateau stress of IEH structures in BD is barely reduced, but it in LD is incredibly enhanced. Therefore, the SEA of IEH is slightly reduced in BD but multiplied several times in LD. The above results and comparison demonstrate that the IEH structures can achieve 64% to 75% of the mechanical properties and energy absorption in LD compared to BD, which is overwhelmingly better than the value of less than 10% for RHH structures.

### 3.3. Crushing Behaviors

**Figure 9:**
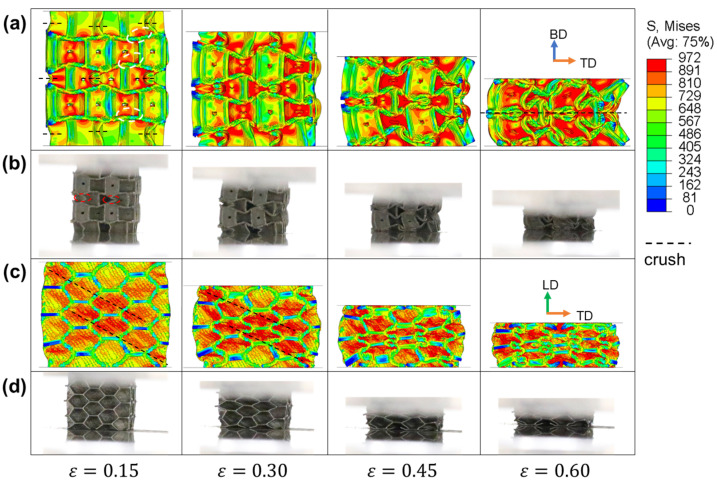
The stress distributions of IEH-g1 and its corresponding photos at various strains: (**a**) front view in FE simulation; (**b**) front view in the experiment; (**c**) side view in FE simulation; (**d**) side view in the experiment. The dashed line represents the location where crushing happens, and the dashed circles mark the initial stress concentration.

To investigate the deformation mode and failure mechanisms of IEH structures under quasi-static compression, the diagrams of stress distribution in various strains from FE simulations corresponding to the in situ photos were plotted. The results of IEH with no gradient (g1) are illustrated in Figure 9. To observe the structures from the front view, it can be found that the odd layer of cells crushes first, especially the middle layer. The half of vertical walls of the hexagonal cells in the middle layer are first crushed to form triangular and inward-folding hexagonal shapes. During the deformation, the middle section of the structure bulges outwards on one side to create an arc shape. It can also be found that the stress concentrates on the transitions between walls in various directions at the initial deformation of structures. Similar geometrical characteristics are also observed from in situ photos in experiments. From the observation on the side view from Figure 9c,d, the deformation characteristics of IEHs are similar to those in RHHs, where diagonal shear bands are formed first and gradually expanded, leading to a synchronous crushing of the overall structure. Furthermore, during the LD compression, the walls of structures oriented perpendicular to out-of-plane direction act as reinforcing ribs, distributing the most of stress, thereby enhancing the in-plane load-bearing performance.

**Figure 10:**
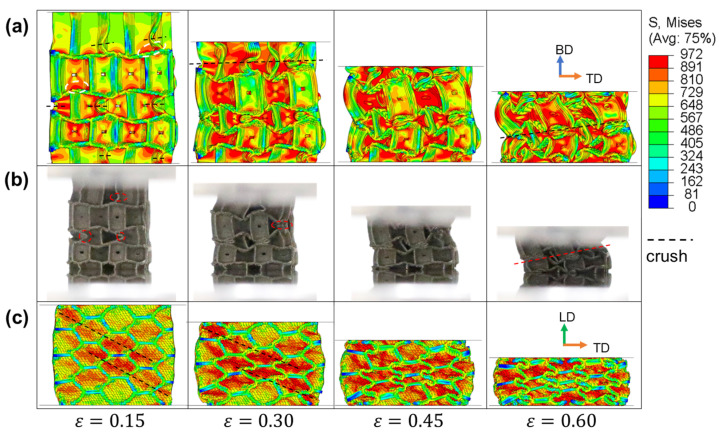
The stress distributions of IEH-g2 and its corresponding photos at various strains: (**a**) front view in FE simulation; (**b**) front view in the experiment; (**c**) side view in FE simulation. The dashed line represents the location where crushing happens, and the dashed circles mark the initial stress concentration.

**Figure 11:**
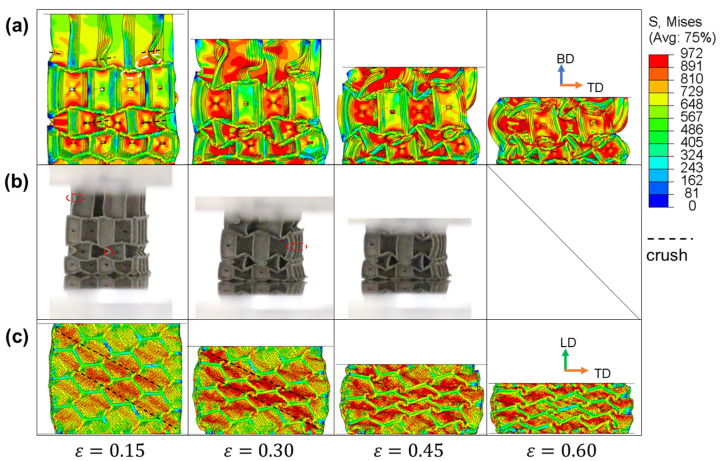
The stress distributions of IEH-g3 and its corresponding photos at various strains: (**a**) front view in FE simulation; (**b**) front view in the experiment; (**c**) side view in FE simulation. The dashed line represents the location where crushing happens, and the dashed circles mark the initial stress concentration.

The numerical and experimental results from IEH-g2 and IEH-g3 are illustrated in Figure 10 and Figure 11, respectively. As a gradient is generated in the building direction, the vertical walls at the top layer of hexagonal structures become taller, resulting in a reduction in deflection, and making this region easier to buckle under external loads. However, the middle layer is still the region that first deforms, induced by the structural design and stress concentration. Meanwhile, it is found that in the later stage of deformation, the shapes of IEH structures with gradient no longer exhibit horizontal symmetry as the non-gradient IEH structure, and the structures collapse diagonally. This characteristic of deformation leads to instability during the energy absorption of structures, further resulting in the decrease in plateau stress and SEA. On the other hand, observing the deformation of structures under loading in LD from the side view, the stress distribution and deformation mode are similar to them in non-gradient structures.

### 3.4. Response Under Instantaneous Impact

**Figure 12:**
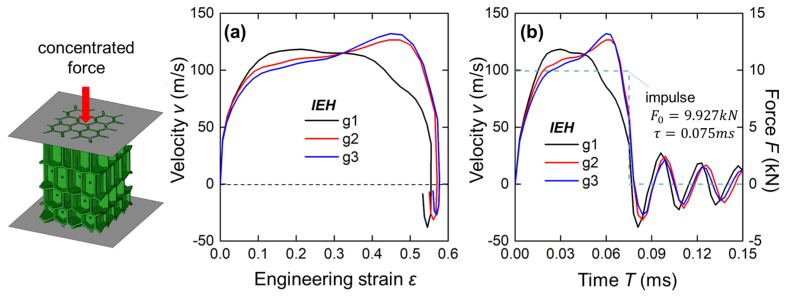
The geometries and boundary conditions in FE simulation and the results of IEHs with different gradients: (**a**) the velocity of the loading plate versus engineering strain of structures; (**b**) the velocity of the loading plate and the concentrated force on the plated versus time.

In this section, simulation cases under blast loading were conducted to analyze the instantaneous responses of IEH structures, as illustrated in Figure 12. Two rigid plates were tied on the tested structures, and a concentrated force with a specific impulse was applied to the plate at the sparse end. As shown in Figure 12b, the concentrated force in a rectangular impulse (initial peak value $F_{0}$ = 9.927 kN, duration time *τ* = 0.075 ms) was applied initially and unloaded afterward. Due to the instantaneous force, the end of structures is accelerated and begins to be crushed. Afterward, the plate enters a stage with a relatively stable velocity with structural energy absorption and then decelerates sharply at the end of the impulse. Moreover, the velocity oscillates around zero after unloading, with a continuous decrease in absolute value. It is noted that the velocity curves of g2 and g3 are different from the curve of g1. The g1 curve obtains high velocity at the early stage of loading, but the velocity is stabilized and gradually decreases until a sharp reduction after unloading. In contrast, g2 and g3 curves are continuously increased throughout the loading period, and then suddenly drop upon unloading.

To evaluate the energy absorption of IEHs under blast loading, the curves of the plate velocity and the engineering strain are presented in Figure 12a. It is found that the non-gradient IEHs show a higher velocity in the early stage of deformation, but decelerate gradually and can fully eliminate the motion speed at a lower strain compared with the other two cases.

## 4. Conclusions

In this work, the isotropy-enhanced honeycomb (IEH) structures were developed and fabricated by the micro-LPBF technique, and the numerical and experimental investigations on these structures were conducted in comparison with regular hexagonal honeycomb (RHH). Their differences in in-plane and out-of-plane performances were thoroughly studied with respect to compressive responses, mechanical properties, crushing behaviors, and responses under blast loading. The following findings are drawn:

1. The IEHs demonstrate a continuous hardening deformation along the out-of-plane direction, whereas the RHHs have a softening deformation. In contrast, both structures maintain a relatively stable plateau stress under the in-plane loading, and the IEHs exhibit significantly higher stress levels compared to the RHHs.
2. In comparison with RHHs, the out-of-plane performances of IEHs in terms of stiffness and strength, especially the elastic modulus and yield stress, are decreased by approximately 37%, but the reduction in average plateau stress is minimal, leading to a slight deterioration (13%) of specific energy absorption (SEA). In contrast, the in-plane performances of IEHs are extremely promoted by around 500%, and particularly, the stiffness is increased by up to 1200%. Consequently, the in-plane strength and energy absorption capability of IEHs have been significantly enhanced, reaching 64% and 75% of the out-of-plane properties, respectively.
3. With out-of-plane loading, IEHs initiate crushing from the middle layer of structures. By introducing layer-to-layer intervals, the geometrical feature of deformation is no longer horizontally symmetric, while under in-plane loading, the diagonal shear bands are formed and the structures undergo entire deformation, where the connecting walls between layers are crucial to dispersing stress concentration.
4. Under an out-of-plane instantaneous impact, the deceleration performance of IEHs with layer-to-layer interval gradient differs from non-gradient IEHs, where the IEH with a larger gradient exhibits better a cushion property at the earlier stage, and the non-gradient one performs better at the later stage.

To sum up, the IEHs exhibit superior improvements in in-plane performances, achieving approximately a 70% comprehensive level of out-of-plane performances, which is substantially better than the ratio of around 10% for RHHs. From an engineering perspective, sandwich panels constructed with IEH as core material offer enhanced resistance to multi-directional impacts, making them well-suitable for more diversified cushioning and impact-resistant requirements.

## Figures and Tables

**Table 1:** Parameters for structures in different design configurations.

Case		Initial Distance *d*_0_ (mm)	Relative Density *ρ_s_*	Shell Thickness *t* (mm)	Distance of Seeds *l* (mm)
λ	1	2.57	0.220	0.2	$\frac{4\sqrt{3}}{3}$
	1.1	1.72	0.218		
	1.2	1.12	0.219		
RHH		\	0.188		

**Table 2:** Process parameter configurations.

Parameters	Value
Laser power	50 W
Laser spot size	25 µm
Layer thickness	10 µm
Scanning speed	1000 mm/s
Hatch spacing	50 µm
Hatch angle	67°

**Table 3:** Material properties of the LPBF-fabricated SS316L bulks.

Direction	Elastic Modulus *E* (GPa)	Poisson’s Ratio *v*	Density *ρ*(kg/m^3^)	Yield Stress *σ_y_* (MPa)	Strength Coefficient *K* (MPa)	Hardening Rate *c*	Fracture Strain *ε_p_*
BD	73.9	0.3	8000	529	836	0.54	0.320
LD				661	681	0.71	0.364

## Data Availability

The original contributions presented in the study are included in the article, further inquiries can be directed to the corresponding author.

## Abbreviations

The following abbreviations are used in this manuscript:

RHH	Regular Hexagonal Honeycomb
IEH	Isotropy-Enhanced Honeycomb
FE	Finite Element
LPBF	Laser Powder Bed Fusion
AM	Additive Manufacturing
SLM	Selective Laser Melting
BD	Building Direction
LD	Lateral Direction
TD	Transverse Direction
SEA	Specific Energy Absorption
